# Tailoring the Input to Children's Needs: The Use of Fine Lexical Tuning in Speech Directed to Normally Hearing Children and Children With Cochlear Implants

**DOI:** 10.3389/fpsyg.2021.676664

**Published:** 2021-06-17

**Authors:** Lotte Odijk, Steven Gillis

**Affiliations:** CLiPS, Department of Linguistics, University of Antwerp, Antwerp, Belgium

**Keywords:** infant directed speech, cochlear implant, word acquisition, language development, mean length of utterance

## Abstract

**Purpose:** The aim of the present study was to explore fine lexical tuning in Dutch infant-directed speech (IDS) addressed to congenitally deaf infants who received a cochlear implant (CI) early in life (<2 years of age) in comparison with children with normal hearing (NH). The longitudinal pattern of parents' utterance length in the initial stages of the child's lexical development was examined. Parents' utterances containing the words the children eventually acquired in the earliest developmental stages were selected and their MLU (Mean Length of Utterance) was measured.

**Method:** Transcriptions of monthly recordings of spontaneous interactions of 10 CI children and 30 NH children with their parents were analyzed. The children with CI were followed from the moment their device was switched on, and the NH children from the age of 6 months onwards. A total of 57,846 utterances of parents of CI children and 149,468 utterances of parents of NH children were analyzed.

**Results:** IDS addressed to children with NH and children with CI exhibits fine lexical tuning: parents adjust the MLU of the utterances that contain the words that children are on the verge of producing themselves. More specifically, the parents' mean length of those utterances decreased in relation to the point when the children began using the item. Consequently, the number of occurrences in isolation of the lexical item increased. The speech addressed to all the children exhibited this phenomenon, but it was significantly more strongly present in speech addressed to the children with CI.

**Conclusions:** The speech addressed to children with NH and CI is characterized by fine lexical tuning and a high incidence of single-word utterances in the period leading up to the children's first use of words in speech production. Notwithstanding striking commonalities, IDS addressed to children with a hearing impairment is markedly different, which suggests that parents take this specific character of the children into account.

## Introduction

Infant Directed Speech (IDS) is a simplified register that adults often use with their infants. The register is characterized by specific adaptations as compared to Adult Directed Speech (ADS), such as shorter and simplified utterances, a higher pitch, a greater variability in pitch, a larger vowel space and a slower speaking rate (Phillips, 1973; Snow, 1977; Fernald et al., 1989; Soderstrom, 2007; Ko, 2012; Cristia, 2013; Wieland et al., 2015).

IDS attracts and maintains the infants' attention, as is shown by infants' preference for IDS over ADS (Cooper and Aslin, 1990; Wang et al., 2018), communicates affect (Benders, 2013) and has beneficial effects for language acquisition in general and lexical acquisition in particular. For example, children who heard more IDS at 19 months old were more efficient in processing familiar words (Weisleder and Fernald, 2013) and the amount of IDS and number of maternal conversational turns appears to be a predictor for the child's expressive vocabulary size (Weisleder and Fernald, 2013; Vanormelingen et al., 2016). Furthermore, IDS supports word learning in the early stages of lexical acquisition. Twenty one-month-old infants only learned novel words when heard in IDS and not when heard in ADS (Ma et al., 2011). However, 27-month-olds learned novel words presented in both IDS and ADS, implying that IDS has a larger influence on word learning in the early stages of lexical development (Ma et al., 2011). Thus, IDS appears to be important for lexical acquisition, especially in the earlier stages of lexical development.

IDS has an influence on the language abilities of children, but the reverse is also true: the abilities and characteristics of a child influence the speech of parents. A characteristic that appears to influence parents' adaptation of their speech is the hearing ability of the child. Several studies have investigated the influence of infants with hearing impairment fitted with a cochlear implant (CI) on parents' speech. The results of these studies show similarities with IDS addressed to NH children, but there are also differences. IDS addressed to hearing impaired children is also characterized by higher pitch, shorter utterances, longer pauses and a larger vowel space compared to ADS [Bergeson et al., 2006; Wieland et al., 2015, but see Lam and Kitamura (2012)]. Moreover, research has shown that IDS to CI infants is more similar to hearing experience-matched NH infants than to age-matched NH infants. For instance, Bergeson et al. (2006) found that pitch and pause duration in IDS was similar when spoken to CI children and hearing experience-matched NH children, but differed with age-matched NH children [see also Kondaurova et al. (2013)]. This suggests that some characteristics of IDS are controlled by the hearing experience of the infants rather than their chronological age (Bergeson et al., 2006; Kondaurova et al., 2013). Some studies report that mothers of hearing impaired infants with an acoustic hearing aid produce fewer utterances than mothers of age-matched NH infants (Lederberg and Everhart, 1998; Clement, 2004). However, VanDam et al. (2012) and Vanormelingen et al. (2016) found that in IDS the amount of input to hearing-impaired children fitted with a CI or hearing aids is comparable to the amount of input to NH children. The parents of CI children and NH children were equally talkative. However, even though the amount of input was similar, mothers used longer utterances and spoke faster when speaking to NH children compared to speech to CI children (Kondaurova et al., 2013; Vanormelingen et al., 2016). Moreover, MLU of utterances directed to CI children was lower than the MLU of utterances directed to their age-matched NH peers (Fagan et al., 2014).

In sum, clear differences and commonalities between IDS to NH children and to CI children have been established. IDS is adapted to the linguistic level of children, as indicated by the larger differences of IDS addressed to age-matched CI children and their NH peers in comparison to children matched on their hearing experience. The differences in parents' MLU, speaking rate and vowel space also imply that the characteristics of a child determine the speech of parents to their children.

### Infant Directed Speech as a Dynamic Phenomenon

If parents fine-tune their speech to the linguistic level of the child, then IDS should be conceived as a dynamic phenomenon that is responsive to the child's evolving abilities. This was first suggested in the fine-tuning hypothesis: IDS is continuously changing to suit the changing needs of the child (Snow and Ferguson, 1977). A characteristic that is adapted to the child's linguistic ability is the mean length of utterance (MLU) in parents' speech (Sherrod et al., 1977; Murray et al., 1990; Ko, 2012). Findings reveal that MLU in IDS changes non-linearly during the first years of a child's life, with a change around the transition from pre-verbal to verbal stage. Parents' MLU decreases at approximately 6 months of age, probably because children begin to show signs of comprehending common words around that age (Bergelson and Swingley, 2012). This type of tuning is called coarse tuning: adjustments of the parents' speech to the general linguistic ability of the child (Roy et al., 2009). Speaking rate is another example of coarse tuning. The speaking rate of the caregiver also shows a non-linear trend: it decreases abruptly around the time children begin to produce their first words and increases again when children enter the multiword stage, showing a U-shaped curve (Ko, 2012).

Whereas, coarse tuning refers to parents tailoring their utterances to the general linguistic ability of the child in a broad sense, fine tuning may also occur at a more fine-grained level, specifically at the level of individual lexical items. Roy et al. (2009) baptized this phenomenon fine lexical tuning, which implies tuning into the child's inferred knowledge of individual lexical items. Parents appear to adjust the complexity of their utterances to the familiarity of the child with particular words (Roy et al., 2009). More specifically, parents appear to systematically use (on average) shorter utterances containing the words that the child is on the verge of acquiring. In a case study, Roy and colleagues analyzed the length of parents' utterances containing particular words. They found a systematic decrease of MLU in IDS in the period of time preceding the “word's birth,” i.e., the first time a word was produced by the child. In a replication study involving 30 typically developing children, a phenomenon similar to the one highlighted in Roy et al.'s (2009) case study was established: a decrease in parents' MLU before word birth and a slight increase months after word birth (Odijk and Gillis, 2021). Thus, parents seem to adjust their utterances when tuning in to the infant's lexical knowledge.

In sum, there seems to be coarse tuning in IDS: the parents' MLU decreases when children start to talk, and even before that, specifically when they show the first signs of word comprehension. The adaption is even more striking in fine lexical tuning, which shows adaption of MLU in IDS at the level of individual words.

### Infant Directed Speech and Word Learning

It is important that children pay attention to speech to learn words. Infants with a CI have a more difficult task because of their struggle to access the auditory input. The first obstacle in this respect concerns attention to speech. CI children have been shown to pay less attention to speech than NH infants (Houston et al., 2003; Horn et al., 2007; Houston and Bergeson, 2014). Houston et al. (2003) found in a direct comparison that CI children did not display as much preference for speech sounds as their hearing age-matched NH peers in a visual habituation procedure. This can be a result of their worse hearing, but may also be a result of their delays in several domains of executive functioning, such as processing speed (Castellanos et al., 2016). However, other recent studies have shown that CI children display similar attention to speech as their age-matched NH peers as early as 3 months post-implantation (Wang et al., 2018).

What is even more important for attention to speech is attention to IDS. Comparisons between IDS and ADS have shown that both NH and CI infants prefer IDS over ADS. However, CI infants, unlike NH infants, show no preference for ADS over silence (Wang et al., 2017). Thus, because of the preference for IDS and the lack of attention to ADS, it seems that IDS is essential for CI children to enhance their attention to speech. Both NH and CI infants pay less attention to ADS than to IDS, but, in addition, children with a CI pay less attention to the details of speech. Research on the words known by CI children has shown, for instance, that they appear to be insensitive to the effect of phonotactic probability, i.e., the likelihood of occurrence of a sound sequence in a word. Usually, children learn words with a low phonotactic probability faster in the initial phase of word learning, because these words can be more easily be recognized as novel words. The lack of phonotactic probability effect in CI children suggests that they are far less sensitive to phonological information, which is hypothesized to lead to less efficient word learning (Han et al., 2015).

Attention to speech, and especially IDS, is beneficial for word learning (Ma et al., 2011). But the ease of word acquisition also depends on the way in which words are presented to children. For instance, infants have more difficulties segmenting words from fluent speech (Mattys and Jusczyk, 2001; Seidl and Johnson, 2006) than words uttered in short utterances or words spoken in isolation, because these words appear to be easier to segment and thus to learn. Indeed, research has shown that words that occur frequently in short utterances in IDS are produced earlier by children (Grimm et al., 2019). Moreover, words uttered frequently in isolation by their parents appear to be easier to acquire. According to Brent and Siskind (2001) the odds of learning a word increase when it is regularly heard in isolation. Swingley and Humphrey (2018) reexamined the data set of Brent and Siskind (2001) but included more predictors in their statistical model, such as the frequency of words in utterance final and other positions. They found similar results: words that were presented in isolation or in shorter utterances, were more often understood and produced by 12- and 15-month-old children. Novel words were also more readily learned when presented in isolation than when presented sentence-finally by 12-month-old infants (Keren-Portnoy et al., 2019). These findings suggest that particular aspects of fine lexical tuning can be useful for language learning. Thus, if words in isolation or in short utterances aid lexical acquisition, it can be expected that in parents' fine lexical tuning, a particular word will occur more frequently in shorter utterances or even in one-word utterances around the time that word is first produced by the child.

In sum, mixed results have been found regarding the attention to speech in CI children relative to NH children. They do not pay attention to ADS, but they do pay attention to IDS. This suggests that IDS is beneficial for their attention to speech. Since CI infants struggle to access the auditory input, they might benefit even more from shorter utterances where the word is easier to segment. Hence, single-word utterances may be especially beneficial to them.

### Word Classes

Is every word type as easy to learn as any other, or are there differences in the acquisition of word classes? It has been repeatedly reported that nouns are learned earlier than verbs. This preference for nouns over verbs is called the noun-bias (Gentner, 1982) and most children show this bias. However, the noun bias is not found in some languages, such as Mandarin (Tardif, 1996) and Korean (Choi and Gopnik, 1995). Nouns are argued to be easier for infants to learn, because their referents are easier to map onto the world than the referents of verbs and other relational terms. For example, a dog can be seen and be pointed at in the world when learning the word “dog.” A verb, however, has a less transparent relation to the world, so that it is more difficult to establish the word-world mapping in the case of verbs (Gentner, 1982). In addition to this conceptual transparency issue, other factors also appear to contribute to an account for the noun-bias. Nouns are more frequent in short utterances and at the end of longer utterances, nouns outnumber verbs in frequency and there is a preponderance of nouns in salient utterance positions (utterance initial and utterance final position) (Goldfield, 1993; Longobardi et al., 2015, 2016). All these factors appear to favor the acquisition of nouns as opposed to verbs. However, these characteristics do not apply to all languages, for example, it is found for Mandarin, where there is no noun-bias, that the grammar and input appears to highlight verbs (Tardif, 1996).

The preponderance of nouns has been attested in the vocabularies of NH children as well as CI children (Le Normand et al., 2003), in typologically diverse languages, though, as indicated before, this pattern cannot be assumed to be a universal trait. Furthermore, CI and NH children show a similar distribution of word categories: nouns are the most frequent, followed by predicates (Nott et al., 2009; Jung et al., 2020). In addition to overwhelming commonalities, there are a few differences in the composition of the vocabularies of the two groups. Nott et al. (2009) found that the proportion of nouns is greater in the lexicon of the NH children than in that of the CI children. The CI children use relatively more predicates and onomatopoeic words. How can the differences between NH and Ci children be explained? Even though both groups exhibit a noun-bias, NH children know proportionally more nouns than CI children (Nott et al., 2009). Differences in the IDS directed at NH and CI children may be relevant in this respect. Here we investigate the possibility that the differences can at least in part be attributed to differences in the frequency of single-word utterances in parents' speech.

### Current Research

The aim of the present study was to explore fine lexical tuning in Dutch infant-directed speech (IDS) addressed to congenitally deaf infants who received a cochlear implant (CI) early in life (<2 years of age) in comparison with children with normal hearing (NH). Previous research investigating IDS addressed to NH and CI children revealed commonalities as well as disparities between the two. As to the latter, differences in the vowel space in IDS to CI children and NH children (Lam and Kitamura, 2012), MLU (Fagan et al., 2014) and speaking rate (Kondaurova et al., 2013; Vanormelingen et al., 2016) have been established. However, pitch, pause duration, and the amount of input of parents to both groups of children were found to be similar (VanDam et al., 2012; Vanormelingen et al., 2016). In the current research two aspects of IDS were addressed: (1) coarse tuning as an adaptation to the child's perceived linguistic sophistication, and (2) fine lexical tuning as an adaptation to the child's mastery of specific lexical items.

The first purpose of the study was to examine the longitudinal pattern of Dutch-speaking parents' utterance length during the first stages of lexical development. The specific question addressed in this respect is whether parents of children with CI implement coarse tuning similar to parents of NH children when infants are starting to use their first words? In order to answer this question, a longitudinal corpus of speech directed to NH children and CI children was analyzed. The MLU of the parents' speech interacting with their children over time was measured. Parents have been shown to be sensitive to the linguistic level of children with NH and to adjust their language accordingly. A similar adjustment of the overall MLU is expected for parents of CI children. More specifically, the MLU of IDS addressed to CI children is expected to be lower than IDS addressed to NH children, given the reports in the literature in which the MLU addressed to CI children was lower than the MLU addressed to NH children (Fagan et al., 2014). But contrary to the dyads reported on by Fagan et al. (2014), who were matched by the infants' chronological age, the children participating in the present study were matched on a measure of their linguistic development, specifically, their cumulative vocabulary. The main reason is that at the same chronological age, CI and NH children have fairly different hearing ages, and hence probably have reached significantly different levels of linguistic development (Bergeson et al., 2006; Kondaurova et al., 2013). Hence, if children are aligned relative to a measure of their linguistic development, differences in their input should probably be attributed to the differences in the children's hearing status.

The second purpose of the study was to address fine lexical tuning in IDS to CI and NH children. It is expected that parents of CI children implement similar changes at the level of individual lexical items as parents of NH children. More specifically, parents of NH children have been shown to adjust their speech at the level of individual words: they shorten their utterances containing the words that the child is on the verge of acquiring (Roy et al., 2009; Odijk and Gillis, 2021). Consequently, in the present study the evolution of the MLU of the IDS addressed to NH and CI children was not aligned on chronological age, but relative to the first appearance of particular words in the child's speech (Odijk and Gillis, 2021). It was expected that caregivers of infants with CI would tune their utterances to the emergence of words even more than caregivers of children with NH, thus showing their inclination to compensate for the children's auditory limitations. In other words, the process of fine lexical tuning was expected to be even more salient in IDS addressed to children with CI than in IDS addressed to children with NH.

Two consequences of fine lexical tuning were further investigated. The first related to the increasing incidence of single-word utterances with a particular word as the moment of the child's first production of that word approached. The second concerned the type of words that occurred in single-word utterances as a possible explanation of asymmetries in the child's initial vocabulary.

If parents use fine lexical tuning, utterance length was expected to decrease as a word's entrance in the child's vocabulary approached. Consequently, the incidence of a word appearing in isolation, that is, as a one-word utterance, was predicted to increase. Moreover, it was expected that CI children might even benefit more from isolated words, as they struggle to access the auditory input (Han et al., 2015). Thus, words in isolation were expected to occur even more frequently in IDS addressed to CI children.

The second consequence of fine lexical tuning that was investigated in the present study related to the types of words occurring in isolation in IDS. If there are more words in isolation as word births approach, and if isolated words are beneficial for word learning, it was expected that the words that occur the most frequently in isolation in IDS, are also the most frequent in children's vocabularies. If nouns can be shown to occur more frequently in isolation in IDS than the other word classes, this observation can serve as an additional explanation of why nouns are more common in the initial vocabularies of children than other word classes.

In summary, the following research questions were pursued in the present research: (1) Do parents of CI children adjust their speech to the perceived linguistic sophistication of the children similar to parents of NH children when infants are starting to use their first words? And (2) how do parents of CI children adjust their speech at the level of individual words compared to NH children? In addition, two further questions relative to fine lexical tuning were investigated: (1) what is the incidence, in one-word utterances, of words the child eventually acquires? And (2) what is the distribution of word classes in the one-word utterances addressed to NH and CI children?

## Method

The data for this study were taken from the CLiPS Child Language Corpus (CCLC), a collection of longitudinal recordings of dyadic interactions. The corpus consists of audio and video recordings of spontaneous speech of Dutch monolingual children, living in Flanders, the Dutch-speaking part of Belgium. The parents of all children were native speakers of Dutch as spoken in Flanders, normally hearing, and from a mid-to-high SES background (Schauwers, 2006; Molemans, 2011; van den Berg, 2012; Van Severen, 2012).

### Participants

The data of ten children with CI and thirty children with NH were extracted from the CCLC database. The children with CI were diagnosed with a congenital hearing impairment, which was confirmed by TEOAEs and/or ABR (neonatal hearing screening) in the first weeks of life. Their medical records and the treating audiological center did not mention any other additional health or developmental problems. All children had a congenitally profound hearing loss with an unaided pure-tone average (PTA) ranging from 93 to 120 dBHL (mean = 113, *SD* = 8.72). One to four months after the detection of their hearing loss, nine of the ten infants were fitted with bilateral acoustic hearing aids. The remaining infant started wearing bilateral hearing aids 8 months after detection of her hearing loss. Since the auditory progress with their hearing aids was deemed insufficient by the multidisciplinary staff of the audiological center (see Table 1), they were enrolled as candidates in a cochlear implant program. All children received a multichannel Nucleus-24 implant between 0:5 (years:months) and 1:08 (mean age 1:0, *SD* 0:05). At 2:0 their PTA had decreased to 28–53 dBHL (mean = 40.10, *SD* = 8.24). Two children received a second implant during the study period. All children were raised orally in Dutch, with the help of a limited number of lexical signs. An overview of the relevant characteristics of each child is displayed in Table 1.

**Table 1:** Characteristics of children with cochlear implants.

					**Age activation**		
**Subject**	**PTA unaided**	**PTA aided HA**	**PTA aided CI at age 2**	**Age HA**	**Age 1st CI**	**Age activation 1st CI**	**Age 2nd CI**
S1	120	120	48	0:9	1:1	1:3	–
S2	120	120	30	0:1	0:6	0:8	–
S3	115	113	33	0:2	0:10	1:0	–
S4	113	117	48	0:10	1:6	1:7	–
S5	93	47	38	0:5	1:5	1:6	–
S6	120	107	53	0:2	0:9	0:10	–
S7	117	107	42	0:4	0:5	0:6	1:3
S8	112	58	38	0:2	1:7	1:9	–
S9	103	63	28	0:5	0:9	0:10	1:11
S10	91↓117	45↓115	43	0:3	1:1	1:2	–

*↓progressive hearing loss*.

A control group of thirty typically developing children were also followed longitudinally. The children were normally hearing with no health and developmental problems according to parental report and the regular observations provided by the Flemish agency *Child and Family* (*Kind & Gezin*). Moreover, during data collection the children's receptive and productive vocabulary development was monitored by administering the N-CDI (Zink and Lejaegere, 2002) at 1:0, 1:06, and 2:0. The results of the testing revealed N-CDI-values within the normal range for all children. The children were monolingually raised in Dutch as spoken in Flanders.

### Data Collection and Transcription

The data collection consisted of longitudinal, monthly recordings at the children's homes. The children with CI were followed from the month the implant was activated, i.e., ~1 month after surgery, until 30 months post implantation. A total of 263 recordings was available. Thirty recordings were not available for every child, since sometimes no recording could take place for personal reasons. The average length of a recording was 62 min (median = 62 min, range = 33–82 min). A total of 57,846 parental utterances in IDS was available (mean = 5,785, median = 6,421, range = 3,015–6,739 utterances).

For the children with NH monthly recordings were collected between 0;06 and 2;00. A total of 570 recordings was available. A recording lasted on average 64 min (median = 63 min, range = 33–114 min). A total of 149,468 parental utterances in IDS was available (mean = 4,982, median = 4,857, range = 2,578–7,142 utterances)

The CI children were followed longer than the NH children. The children with CI were followed up to 30 months after their implant was activated, while the NH children were followed for exactly 18 months, i.e., from 6 to 24 months of age. The starting age for the CI children differed, because not every child was implanted at exactly the same age (see Table 1 for the exact ages).

Audio and video recordings were made each month in the children's home environment. The parents were asked to interact normally with their children during the recordings. This resulted in unstructured spontaneous parent-child interactions. For example, parents played with their children, took them outside or had a meal together. The researcher, who was always present at the recording, did not actively engage in the dyadic activities on her own initiative.

After each visit to a child's home, the researcher who was present at the recording made a selection of 20 min for transcription and coding. This selection was done in order to keep the time required for transcription within reasonable limits [see Molemans (2011) for an assessment]. The researcher aimed at selecting parts of the recording in which the child was the most vocally active. Care was taken not to interrupt interactions in the selection process. Long pauses, talk between the parent and the researcher, and noisy parts were avoided. This resulted in multiple sequences of interactions which together made a 20-min recording.

The transcription was made using CHILDES' CLAN program according to the CHAT conventions (MacWhinney, 2000) by the researcher present at the recording session. The utterances of the parents and children were transcribed orthographically and phonemically with stress marking. Children's target words, i.e., the adult equivalent of each word, were also transcribed phonemically. Only adult's utterances directed to children (IDS) were transcribed and analyzed. Utterances addressed to others were marked in the transcripts as “www” according to the CHAT conventions.

Words were identified using the procedure proposed by Vihman and McCune (1994). A word was identified when at least three of the following criteria were met: first, multiple criteria based on context—e.g., determinative context, maternal identification and/or multiple use of the vocalization. Second, multiple criteria based on the shape of the child's vocalization: is the vocalization the (exact) match or prosodic match of the target form. Third, multiple criteria based on the relation to other vocalizations, such as imitation of the vocalization, invariant production and/or the appropriate uses.

The reliability of the orthographic transcriptions of the adults in the NH corpus was checked for interrater and intrarater reliability. For the interrater reliability check, 10% of the corpus was orthographically retranscribed by a second transcriber. Two aspects of the original and the retranscribed transcription were compared: the content (identical words) and the length of the utterances. The percentage of agreement for the content was 82% and for the utterance length 91%. For the intrarater reliability check, the original transcriber retranscribed 5% of the corpus. These transcriptions were compared in the same way as for the interrater reliability check. This resulted in 88% agreement for the utterance content and 93.5% for utterance length (Molemans, 2011; van den Berg, 2012; Van Severen, 2012).

### Language Measures

The expressive cumulative vocabulary of each of the children was compiled using the CLAN software (MacWhinney, 2000). This was accomplished in an incremental way. First, all the different lemmas that a child actually used in the first recording session were listed. Then all the lemmas that were introduced in the following sessions were added consecutively to the child's cumulative expressive vocabulary (henceforth: cumulative vocabulary). In this way, the cumulative vocabulary of each child contained the set of word lemmas that occurred in the child's actual language use and the age at which each word entered the child's vocabulary according to the transcripts. The first use of each word was called the “word birth.” Hence, a child's cumulative vocabulary indicated for each word the age of its “word birth.” Only the content words (nouns, verbs, adjectives and adverbs) were kept for this study.

In Appendix A, an overview is provided of the cumulative vocabulary of the CI children (“CI corpus”) and the NH children (“NH corpus”). For each individual child, the number of lemmas in his/her cumulative vocabulary is indicated according to the child's chronological age (in months). Shaded cells in the table indicate that there was no recording session in that month. The table in Appendix A shows that child (S1), a child with CI, was first recorded at the age of 14 months. The first 9 words were detected in the transcript of the recording made when the child was 21 months old. The recordings continued until the child was 33 months old and at that age the child's cumulative vocabulary contained 351 words. It can also be inferred from the tables that the age range covered for the NH children was from 6 to 24 months exactly, while for the children with CI age ranges differed because of the different ages at which the children received their CI device. In the final transcripts, the actual cumulative vocabularies of the children also show marked differences. For the purpose of the present study, the transcriptions were analyzed relative to the children's cumulative vocabularies up to 250 words. When a child did not reach this limit at the end of data collection, all transcriptions were analyzed. If a child's cumulative vocabulary exceeded 250 words, the transcription in which the child's cumulative vocabulary reached the 250 words mark was the last included in the study. The additional transcriptions were left out of the study. The practical result of this limitation was that almost all the transcriptions of the NH children were used in the analyses, thus including the data from age 0;6 up to age 2;0. Since the ages at implantation of the children with CI as well as the pace of their lexical development differed, the CI children's ages at the start of the analyses actually ranged from 0:6 to 1:8, and at the end the CI children's chronological ages ranged from 2:5 to 3:6 (see Appendix A).

A total of 5,375 word births were identified for the NH children and 2,109 word births for the CI children. The mean age of the NH children was 1:1, 16 when they used their first identifiable word (median = 1:1.7, range = 0:11.0–1:4.2). The CI children were older when they produced their first word. Taking into account the age of implantation, CI children produced their first word on average 5 months and 6 days after implantation (median = 0:6.8, range = 13 days before implantation to 9 months and 6 days after implantation). The NH children had on average a cumulative vocabulary of 179 words (median = 197, range = 56–247) at the age of 24 months and the CI children a mean cumulative vocabulary of 211 words (median = 234, range = 19–246) at the end of the period studied, which ranged from 2:5 to 3:6, since the age at implantation as well as the pace of their lexical development differed. The children's cumulative vocabularies were categorized according to their word classes, resulting in a total of 132 adjectives, 128 adverbs, 871 nouns and 239 verbs that appeared in the CI children's speech and a total of 218 adjectives, 299 adverbs, 2,507 nouns and 554 verbs for the NH children. An overview is provided in Table 2.

**Table 2:** Distribution of content words in IDS.

	**Adjectives**		**Adverbs**		**Nouns**		**Verbs**	
	**No**.	**%**	**No**.	**%**	**No**.	**%**	**No**.	**%**
NH	218	6	299	8	2,507	70	554	15
CI	132	10	128	9	871	64	239	17

The first dependent variable in the statistical analyses was the MLU in IDS. The MLU was measured in two ways: (1) the MLU of all the utterances of the parents (henceforth: the global MLU) and (2) the MLU of the utterances that contained the words present in the children's vocabulary. The global MLU was computed on the entire transcript in each month using the CHILDES software package CLAN. The second measure involved more intricate computation, for which a Python script was written. For each word in the child's cumulative vocabulary, the parents' IDS was scanned chronologically for utterances containing that word. The MLU was then calculated for each month for each word by dividing the number of words by the number of utterances. This led to a time series of MLUs for each word for each child's IDS. The time series were centered around the time the word was first produced by the child. In other words, the month a word was first produced, was denoted as month zero (month 0) for that word. The month before the child's first production of the word was month −1, and the month after the first production of the word was month +1, etc. In the end, this process yielded 18,684 data points of MLU in speech directed to NH children, and 6,849 data points of MLU in speech directed to CI children.

The second dependent variable that was used in the analyses was the frequency of isolated words. For this measure, all the utterances in IDS containing no more than a single word were extracted from the corpus. From this set, only the words that were eventually acquired by the children were selected. Then, for each child, the frequency of isolated words was calculated for each word class per month from word birth.

### Statistical Analysis

The software R (R Core Team, 2018) and the R library *lme4* (Bates et al., 2015) were used to perform multiple linear mixed effect analysis. To answer the first research question, the global MLU was analyzed. For the global MLU, a linear mixed effects analysis of the relationship between global MLU and time from word birth was conducted. Multilevel modeling was used to assess the development of MLU, with child as random effect. As fixed effects, cumulative vocabulary, a quadratic term of cumulative vocabulary, hearing status (CI or NH) and possible interactions between two effects were entered. As random effect, intercepts for each child were added. As such, it was assumed that the children possibly differed at the level of the intercept. A quadratic term of cumulative vocabulary was added, because a scatter plot revealed a curvilinear relationship.

To answer the second research question, the MLU of the utterances that contain the words present in the children's vocabulary and the frequency of isolated words were analyzed. First, a linear mixed effect analysis of the relationship between MLU and time from word birth was conducted. As fixed effects, linear, quadratic, and cubic effects of time, measured in months from the child's first production of a word (word birth), the child's hearing status (CI or NH), the word class of the target word (noun, verb, adverb or adjective) and the child's cumulative vocabulary (without interaction term) were entered. As random effects, intercepts for each child and each word were added, as well as by-subject and by-item random slopes for the effect of time, as measured in months from word birth. The latter were added because it can readily be assumed that children/parents and words differ at the level of the intercept and that the effect of time differs between the children/parents and the different words. There were no obvious deviations from homoscedasticity or normality revealed from visual inspection of residual plots. The quadratic and cubic effects of time were added, because it was expected that the relationship between time and MLU is curvilinear.

Second, the frequency of words in isolation was analyzed. For the multilevel model for frequency of words in isolation, log base 10 frequencies were used. This was done because the frequency of occurrence was skewed. Raw frequencies will be reported in the examples. Thus, the dependent variable was the log frequency of isolated words. Since the dependent variable is continuous, we use a linear mixed effect model. As fixed effects, linear, quadratic and cubic effects of time, as measured in months from word birth, the child's hearing status (CI or NH), the word class (noun, verb, adverb or adjective) and possible interactions between two effects were added. As random effects, we had intercepts for each child and a by-subject random slopes for the effect of time.

The models were built step by step by adding the random and fixed effects one by one. At each step, a likelihood ratio test was used to assess if the effect improved the model. The cut-off level of significance for the analysis was set at *p* = 0.05. Eventually, the best-fitting model was used to determine which effects were significant predictors. This method was based on Bates et al. (2015) and Baayen (2008). The results of the best-fitting model are reported in the next section. For the data analyses, the MLU of words from 17 months before word birth, to 9 months after word birth were analyzed. The other months were excluded, because there were too few data points (*N* < 100).

## Results

The present study addressed two main research questions concerning parents' tuning into their children's linguistic development as reflected in particular aspects of infant directed speech (IDS). First of all, coarse tuning was analyzed in IDS addressed to children with different hearing characteristics, specifically, congenitally deaf children with a cochlear implant and children with normal hearing. Did parents adjust their speech to the perceived linguistic sophistication of the children in both groups? This was investigated by relating the parents' MLU to the children's evolving cumulative vocabulary. And secondly, with respect to fine lexical tuning: did parents adjust their speech at the level of individual words? And more specifically, did they shorten their utterances containing the words in the period leading up to the words' first appearance in the child's speech, the so-called “word birth?”

### Parents' Coarse Tuning Relative to Children's Cumulative Vocabulary

**Figure 1:**
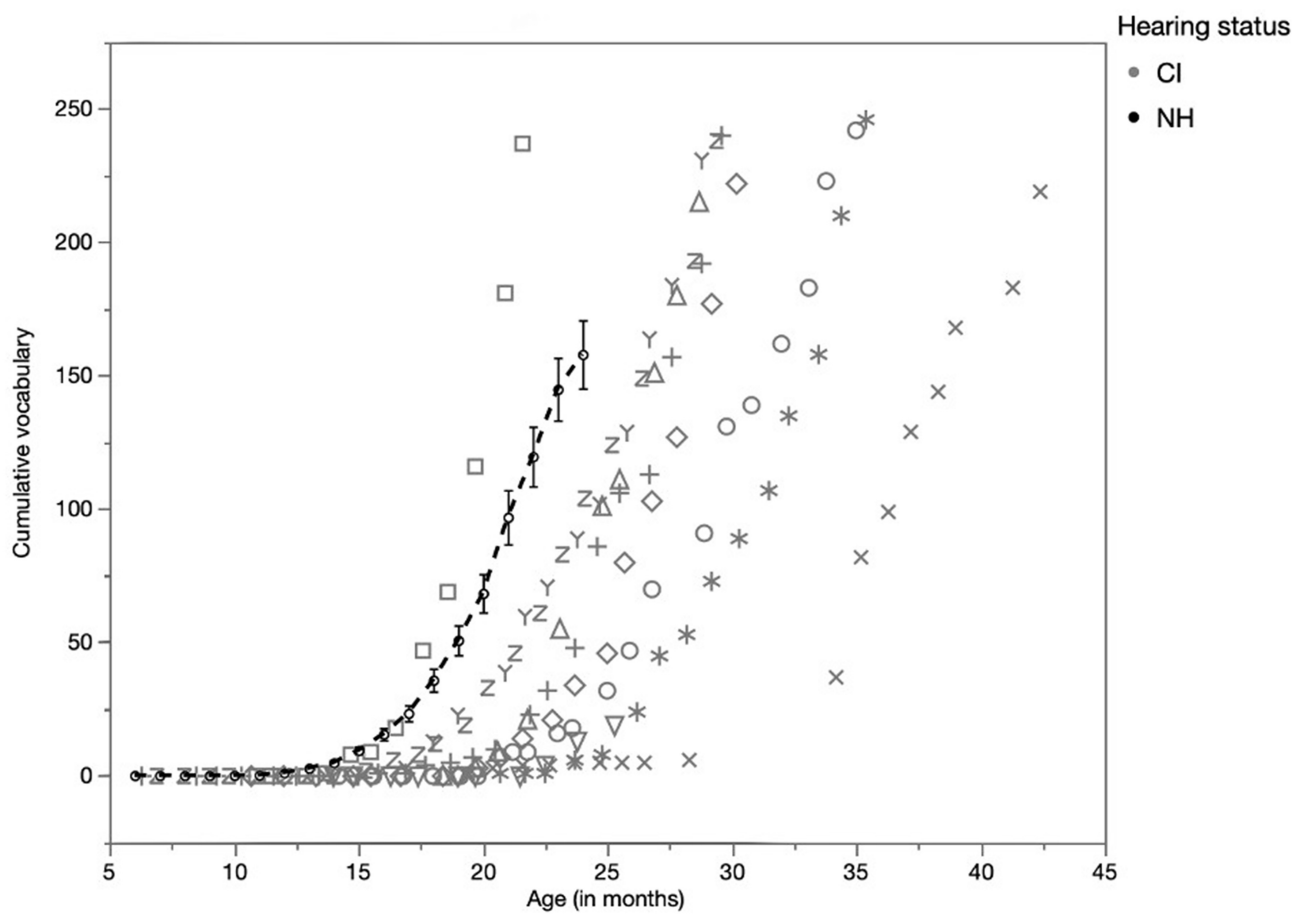
Development of cumulative vocabulary of the NH children and the individual CI children.

In a first instance, the children's cumulative vocabularies were constructed. For this purpose, the transcriptions were analyzed up to the point where the children had 250 words in their cumulative vocabularies. The 250 word boundary was set partly arbitrarily, but also taking the limitation of the data collection into account. Figure 1 shows the development of the cumulative vocabulary of the children. Their individual cumulative vocabulary counts are tabulated in Appendix A. The Figure 1 shows the mean development of the cumulative vocabulary of the NH children with 95% confidence intervals. The curve for NH children stops at 24 months, because there were no recordings after that age. For the CI children, the individual vocabularies of the CI children (as separate symbols) are displayed. One CI child did not reach a cumulative vocabulary of 250, because there was no data collection for this child after 2 years of age (12 months after implantation) due to withdrawal from the study. The graph shows that most CI children were slower in acquiring words than NH children. It takes them more time to acquire the same number of words as NH children do in the same amount of time. Note that there was a 5-month break in the recordings of one CI child around 28 months (the x symbol) due to personal reasons, which probably explains his slower cumulative vocabulary development depicted in Figure 1.

**Figure 2:**
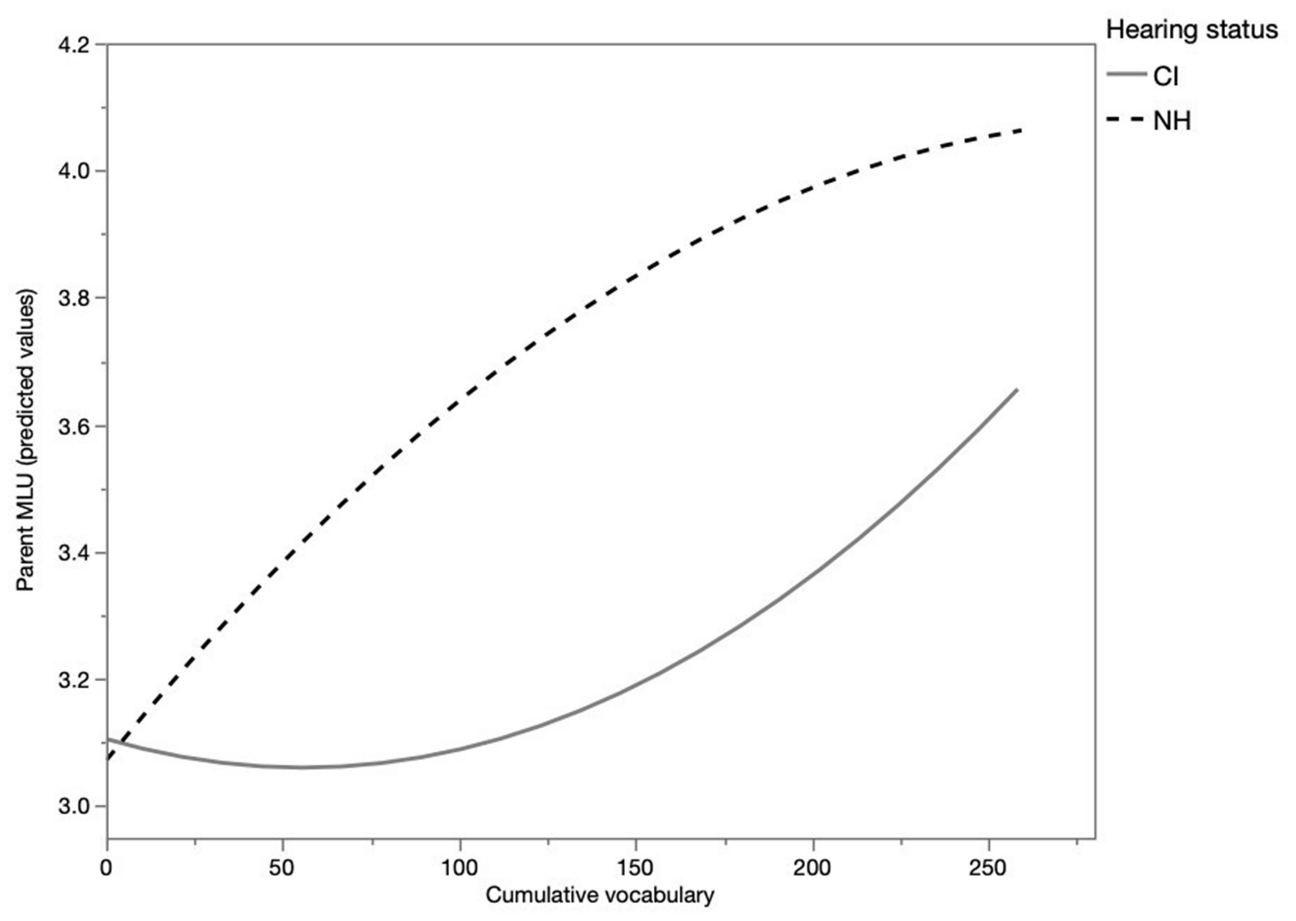
Development of global parent MLU for CI and NH children (predicted values).

How do parents adapt their MLU to the growing linguistic sophistication of the infants, as represented by their cumulative vocabulary? And do parents of CI children adjust their language in a similar way as parents of NH children do? This was assessed by considering the development of their MLU relative to the cumulative vocabulary of the children. For this purpose, the parents' MLU was computed per monthly observation session. Multilevel modeling was used to estimate the development of MLU, with child as a random effect. Table 3 shows the results of the analysis, and a graphical representation of the development of MLU relative to cumulative vocabulary is displayed in Figure 2. MLU increases with increasing cumulative vocabulary of the child as is indicated by a significant effect of cumulative vocabulary (*p* < 0.0001). Moreover, the development is not linear, as shown by a significant quadratic effect of the cumulative vocabulary (*p* < 0.0001). The results reveal no significant effect of hearing status, meaning that there is no statistically significant difference between the MLU of parents of NH children and parents of CI children at a cumulative vocabulary of 0 (the intercept) (*p* = 0.89). However, there was a significant interaction between hearing status and cumulative vocabulary (*p* < 0.05) and hearing status and quadratic cumulative vocabulary (*p* < 0.05). This means that there is a significant developmental difference relative to cumulative vocabulary as can be inferred from Figure 2: the increase of MLU in IDS addressed to NH children is significantly higher than the increase of MLU in IDS addressed to CI children.

**Table 3:** Fixed effects results of the global MLU of IDS directed at the CI and NH children [(CI) = reference category].

	**Estimate**	**SE**	***t*-value**	***p***
Intercept	3.08	0.087	35.69	<0.0001
Cumulative vocabulary	0.008	0.001	7.99	<0.0001
Quadratic cumulative vocabulary	−0.00002	0.000005	−4.03	<0.0001
Hearing Status [CI]	−0.024	0.17	−0.14	0.89
Cumulative vocabulary* hearing status [CI]	−0.006	0.002	−2.76	0.005
Quadratic cumulative vocabulary* hearing status [CI]	0.00002	0.00001	2.23	0.026

### Parents' Fine-Lexical Tuning

**Figure 3:**
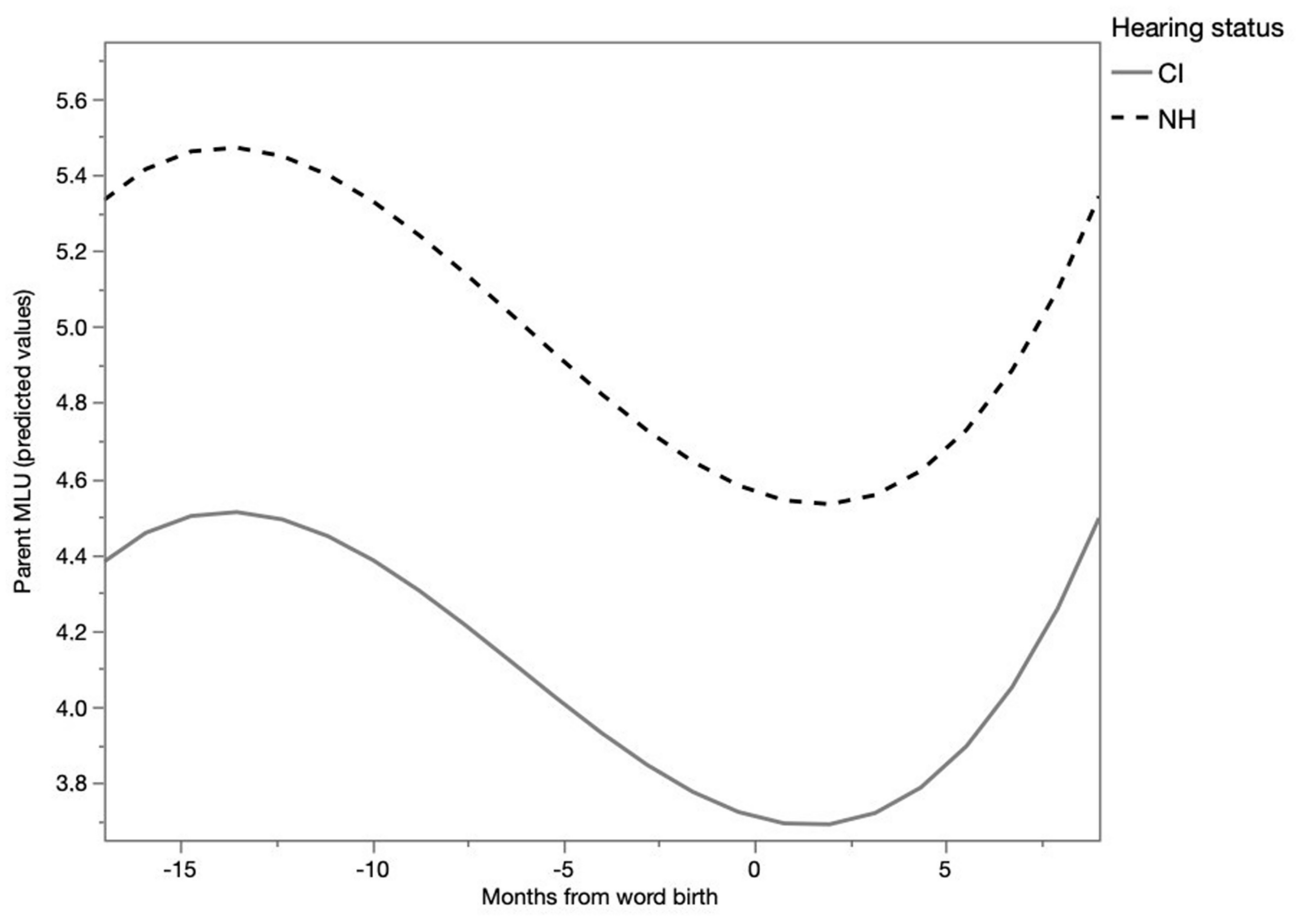
Development of parent MLU according to word birth (predicted values).

The fine-lexical tuning hypothesis predicts that adults' MLU in the utterances in which particular words occur decreases as the birth of those words approaches. Hence, the question addressed here is: do parents tune their utterances to the emergence of words in infants' speech? A multilevel model was fit with random effects for each child and each word for the linear effect of time resulting in random intercepts and slopes for each child and each word. The corresponding model is depicted in Table 1 of Appendix B. The model revealed a statistically significant difference between the NH and the CI group (E = 0.64, SE = 0.23, *t* = 2.83, *p* < 0.05). A Tukey's HSD *post-hoc* test showed that parents of NH children used significantly longer utterances (*M* = 4.93, SE = 0.13) than parents of CI children (*M* = 4.30, SE = 0.21) (*p* < 0.05). Furthermore, there was a significant linear (E = 0.14, SE = 0.03, *t* = 4.99, *p* < 0.0001), quadratic (E = −0.02, SE = 0.002, *t* = −8.21, *p* < 0.0001), and cubic (E = 0.0005, *SE* = 0.00005, *t* = 9.21, *p* < 0.0001) main effect of months from word birth for both groups, explaining the U-shaped curve seen in Figure 3. The utterance length decreased as a word birth was approaching and increased again afterwards. This suggests that parents adapt their utterance length to the emergence of words. In addition, the fixed effect of cumulative vocabulary was significant for both groups (E = 0.001, SE = 0.0003, *t* = 3.30, *p* < 0.001), indicating that parents of NH and CI children increased their utterance length as the child acquired a larger vocabulary, regardless of the time of word birth, thus corroborating the results of the analysis of the global MLU in coarse tuning. An interaction between cumulative vocabulary and hearing status did not improve the model and, hence, was not included in the final model. This lack of interaction indicates that the effect is similar for both groups, which can be inferred from the analysis of coarse tuning seen in Figure 2. This figure shows that both groups of parents increase their MLU with increasing cumulative vocabulary. An interaction between months from word birth and hearing status did not significantly improve the model, so this interaction was not included in the final model. This indicates that the development of MLU depicted in Figure 3 was similar for both groups of children.

**Figure 4:**
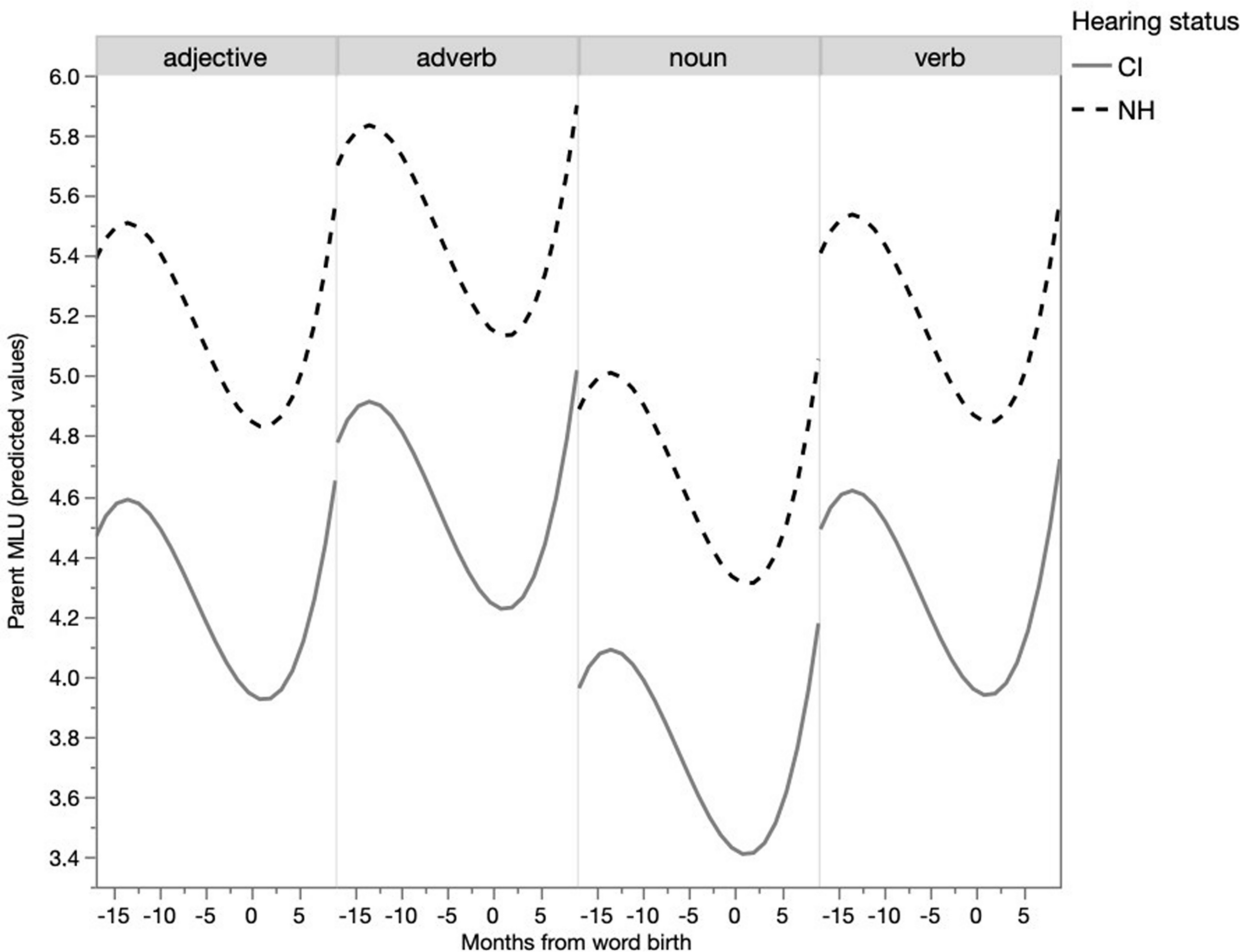
Development of parent MLU per word class (predicted values).

From the previous analysis it appears that parents use shorter utterances with particular words as their children appear to be acquiring them, as indicated by their children's first use. Is this a general, undifferentiated effect or more salient for some word classes? When the word classes were entered into the model reported in Table 1 of Appendix B, a significant main effect for word class was found (*p* < 0.05). The development of utterance length per word class is depicted in Figure 4. A Tukey's HSD *post-hoc* test revealed a significant difference in MLU per word class. Sentences containing target verbs (*M* = 4.70, SE = 0.14), adjectives (*M* = 4.56, SE = 0.18) and adverbs (*M* = 5.03, SE = 0.19), were significantly longer than sentences containing target nouns (*M* = 4.17, SE = 0.12) (p < 0.05). The MLU of sentences with target verbs, adverbs and adjectives were not statistically different from each other. An interaction of the fixed effects word class and months from word birth did not improve the model, and thus was not added in the report of the final model, indicating that the development of MLU was similar for all word classes.

Taken together, parents of NH children used on average more words per utterance when talking to their children than parents of CI children. The MLU of parents of CI children was lower during the entire period studied. Furthermore, the MLU of both parents of NH children and parents of CI children showed a similar development over time, indicating fine-tuning centered around the first appearance of particular words. In addition, the higher the child's cumulative vocabulary, the higher the parents' MLU. Lastly, word class had an effect on utterance length, as sentences with target nouns were shorter than sentences with other word classes.

### Words in Isolation

The results reported in the previous section showed that the MLU decreased as word birth approached. From this reduction of MLU in IDS, it can be inferred that as MLU decreases, single-word utterances increase in frequency. In order to verify this inference, the frequency of words in isolation was mapped relative to the time of word births. It was expected that as word births approached, those words would occur more frequently in parents' single-word utterances. First, the occurrence of words in isolation was calculated and displayed in Table 4. As some word classes were more frequent in our data set than others, it was also measured how often words occurred in one-word utterances relative to the total amount of utterances of target words per word class. Differences between word classes were indeed apparent: nouns were the most likely to occur in isolation: of the 53,947 utterances with target nouns, 6,927 of these utterances were words in isolation (12.8%). Adjectives were a close second (11.3%). Verbs occurred the least in isolation (3.0%). The frequency of adverbs as isolated words was higher than the frequency of adjectives. However, parents did not use many adjectives overall, at least not the adjectives that were present in the child's vocabulary. In relation to the overall use of adjectives, these words were relatively more often spoken in isolation than adverbs in Dutch.

**Table 4:** Proportion of occurrence of words in isolation as a function of word class.

	**Adjectives**	**Adverbs**	**Nouns**	**Verbs**	**All**
Utterances	5,116	31,484	53,947	49,008	139,555
Isolated words	578	2,347	6,927	1,448	11,300
	11.3%	7.5%	12.8%	3.0%	8.1%

**Figure 5:**
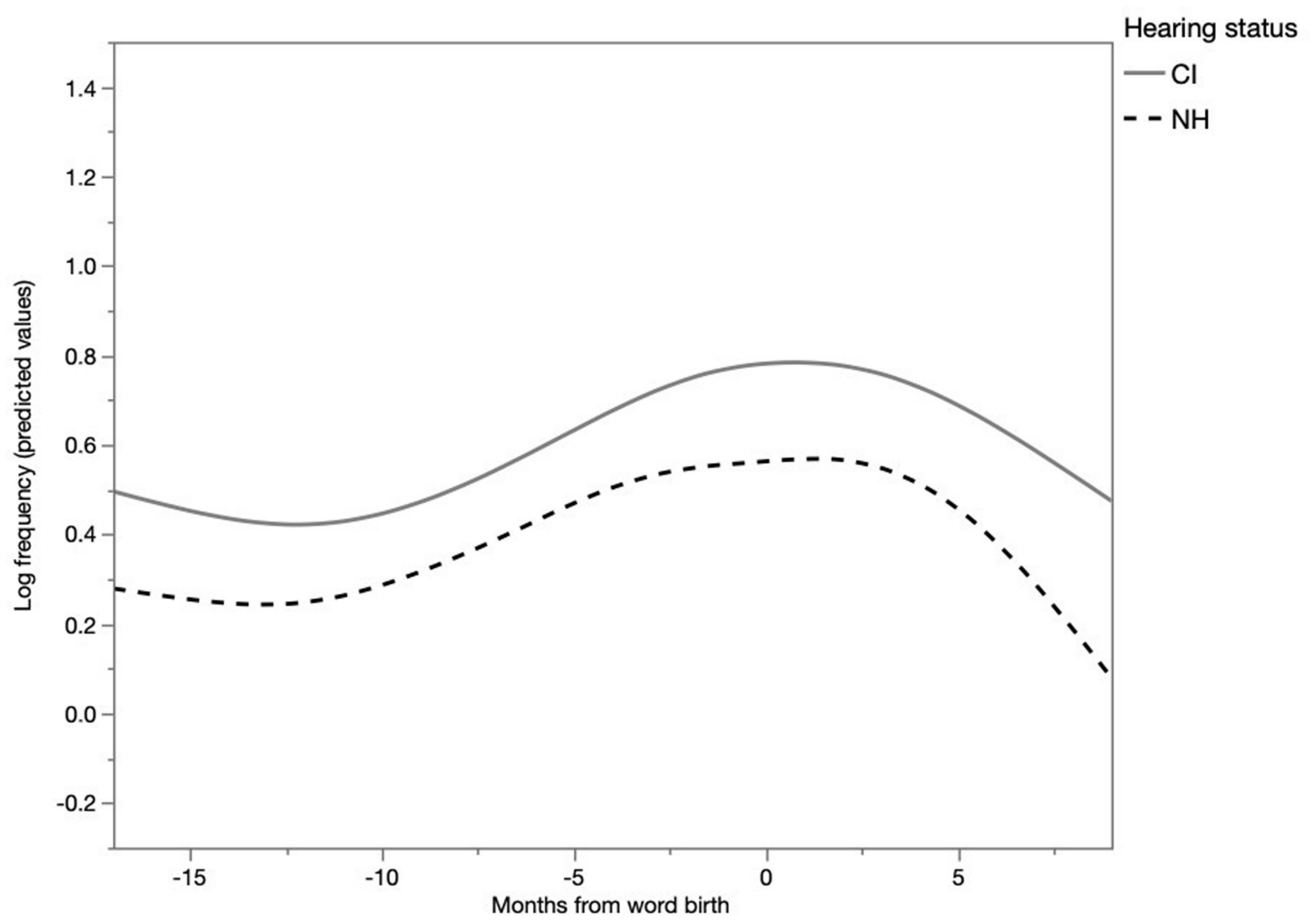
Log frequency of isolated words in IDS (predicted values).

**Figure 6:**
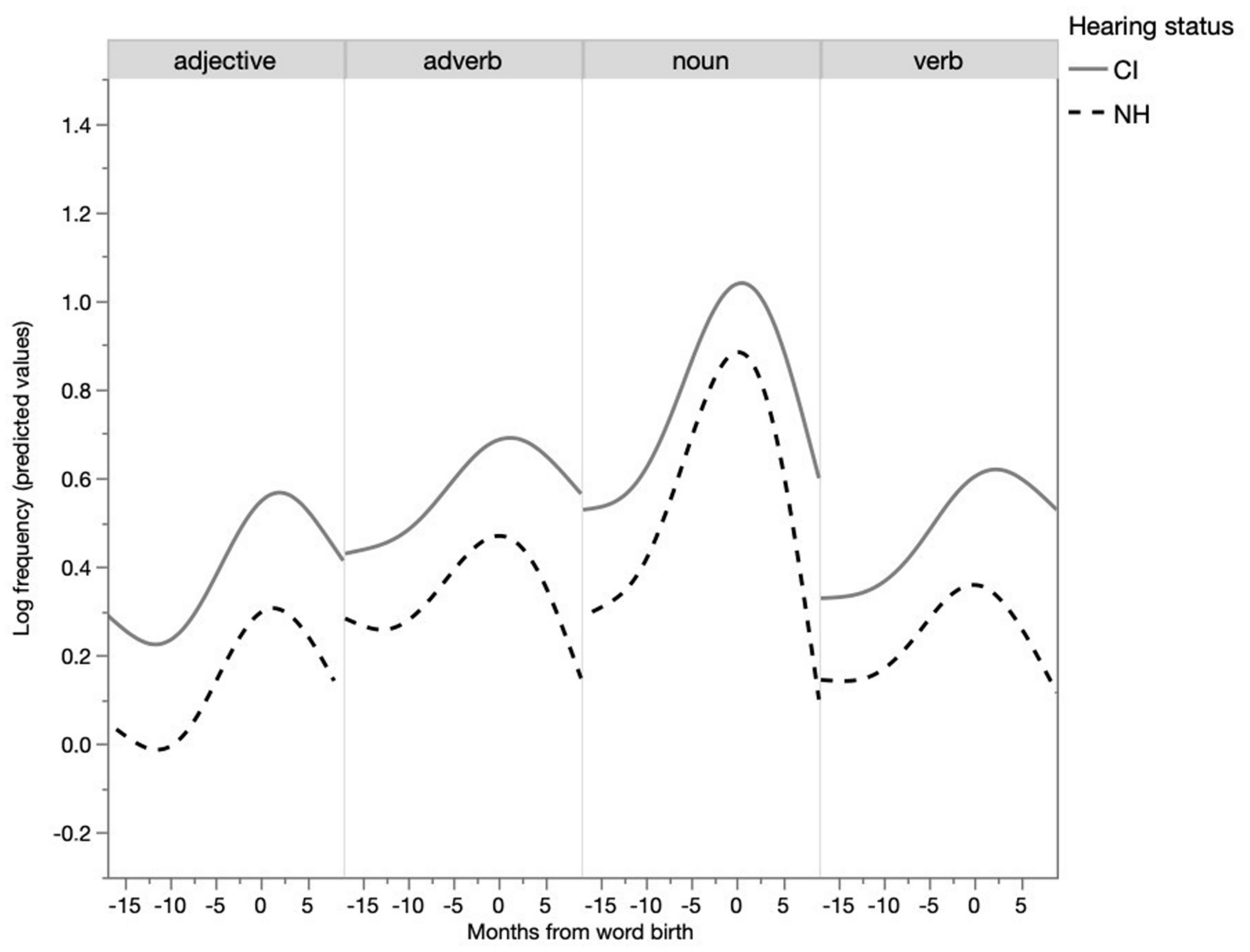
Log frequency of isolated words in IDS per word class (predicted values).

To examine the frequency over time, a multilevel model was fitted with a random intercept and slope for each child each month before and after the child's first production of the word. The corresponding model is tabulated in Table 2 of Appendix B. The first effect was that the more word birth approached, the more frequently a particular word occurred in isolation in IDS, as can be inferred from the significant main effect of months from word birth (linear: E = 0.79, SE = 0.12, *t* = 6.53, *p* < 0.0001, quadratic: E = −0.14, SE = 0.03, *t* = −4.73, *p* < 0.0001, cubic E = 0.02, SE = 0.002, *t* = 8.01, *p* < 0.05). This effect, as depicted in Figure 5, was particularly notable around word birth and immediately after it. Furthermore, there was a significant main effect of hearing status (E = −0.15, SE = 0.04, *t* = −3.63, *p* < 0.001). This indicates that parents of CI children were more likely to use words in isolation than parents of NH children. Moreover, nouns were most likely to occur in isolation, then adverbs, then verbs and least frequent were isolated adjectives, as indicated by a significant main effect of word class (*p* < 0.05). Furthermore, significant interactions were found between word class and linear, quadratic, and cubic months from word birth (*p* < 0.05). This suggests that the development over time of the word classes significantly differed, as can be seen in Figure 6. A Tukey's HSD *post-hoc* revealed that most of the isolated words were nouns (*M* = 0.72, SE = 0.03). Adjectives were the least spoken in isolation (*M* = 0.22, SE = 0.04). In between were verbs (*M* = 0.34, SE = 0.03) and adverbs (*M* = 0.44, SE = 0.28).

In sum, the closer the child gets to word birth, the more parents use isolated words. The frequency of isolated words differed per word class, as nouns were the most occurrent in isolation and adjectives were the least frequent. However, when looking at the proportion of occurrence in isolation, verbs were the least likely to occur in isolation, as only 3% of the utterances with verbs were single-word utterances. In relation to the total utterances with adjectives, adjectives were more often spoken in isolation (11.3%). Furthermore, the development of the MLU of utterances with nouns seems the most notable: it shows the most pronounced U-shaped curve.

## Discussion

The purpose of the present study was to address two main research questions: (1) do parents of CI children adjust their speech to the perceived linguistic sophistication of their children just as parents of NH children when infants are starting to use their first words? And (2) how do parents of CI children adjust their speech at the level of individual words compared to NH children?

### Parents' Coarse Tuning

To explore the first purpose of the study, parents' MLU during the first year of their infant's life was analyzed. The results showed that the utterance length of Dutch-speaking parents of both NH and CI children started at the same level and gradually increased relative to the growing cumulative vocabulary of their children. Is this increase similar for CI and NH children? The development of the utterance length in IDS differed significantly between parents of NH children and parents of CI children: the development of the MLU of parents of NH children increased more rapidly. Thus, the utterance length in Dutch IDS appears to be influenced by the hearing status of the children. This finding agrees with studies showing that mothers of NH children produce more syllables per utterance than mothers of CI children in IDS, in which the children were age matched (Kondaurova et al., 2013; Vanormelingen, 2016). One possible explanation for this difference is that the linguistic development of CI children starts at a slow pace, as seen by their slower development of cumulative vocabulary and slower increase MLU (Faes et al., 2015) than NH children, which could cause parents to adjust their MLU less rapidly. However, our findings contrast with those reported by Bergeson et al. (2006), who found no significant differences between mothers of NH children and mothers of CI children in the number of words per utterance. A possible reason for these discrepant findings could be that Bergeson et al. (2006) matched the children by hearing age and by chronological age, but not by vocabulary size, as was the case in the present study. There was also a difference in the nature of the language samples: Bergeson et al. took their samples of the CI and NH children at one particular point in time, while in the current study the development of MLU was traced over a longer period. Consequently, Bergeson and colleagues were much more dependent on the hazards of a single speech sample, which was far less the case in the present study in which various language samples over time were collected of each mother-child dyad.

In conclusion, our results showed that the parents of CI children and NH children implement coarse tuning, even though the degree of adaptation differs. This implies that Dutch-speaking parents are sensitive to the linguistic development of their children. They increase the complexity of their speech as the linguistic development of the children progresses. Our results showed that the size of the cumulative vocabulary appears to play a role: as vocabulary increases, and as the child's linguistic abilities increase, parents also use more complex language. This partly explains the different path of MLU in CI children: their vocabulary develops much more slowly than that of NH children, and the increase of their MLU is also slower. However, there appears to be an additional effect: at equal levels of cumulative vocabulary, the MLU of parents of NH children is higher than the MLU of parents of CI children. This implies that Dutch-speaking parents adapt their speech not only to the perceived linguistic sophistication of their children, but also to the hearing characteristics of their children. Hence, with an equal level of children's cumulative vocabulary, parents' MLU in addressing children with CI are significantly lower than the MLU of parents addressing children with NH.

### Parents' Fine Lexical Tuning

The second purpose of the present study was to address fine lexical tuning in IDS to CI children and NH children. How do parents of CI children adjust their speech at the level of individual words compared to NH children? Our results demonstrated that parents of NH children and parents of CI children showed a decrease in the length of the utterances containing a word that the child is on the verge of using in production. The utterance length increased again afterwards. Does fine-lexical tuning in IDS occur for CI children similarly to the way in which it occurs for NH children? The development appeared to be similar for both parent groups, however the overall MLU of parents of CI children was lower than the MLU of parents of NH children. This is accordance with our findings for coarse tuning: parents of CI children used shorter sentences in IDS than parents of NH children. This finding suggests that parents take into account the hearing status of their child. They simplify their language in IDS, parents of CI children more so than parents of NH children. Other research has also already demonstrated that parents of CI children produce shorter utterances: they use fewer syllables per utterance in their speech (Kondaurova et al., 2013; Vanormelingen, 2016).

Previous research implied that IDS is more important for word learning in the early stages of lexical development than in the later stages (Ma et al., 2011). This suggests that the reduction of MLU in IDS may be expected to decrease when the lexical abilities of the children grow, as children need less and less scaffolding. The results of the present study corroborate these findings as there appears to be a significant influence of cumulative vocabulary: the higher the cumulative vocabulary, the higher the MLU in Dutch IDS. This indicates that parents increased their utterance length as the vocabulary of their children grew.

In sum, to address the second research question of the study: our results show that Dutch-speaking parents fine-tune their speech to the emergence of words in children and that this is the case for both parents of CI children and parents of NH children. It could be that parents use fine lexical tuning as (implicit) learning strategy. Parents may have indications that the child is responsive to and, hence, may understand specific words, and they adapt their speech accordingly. However, parents may simply mirror the utterances of the children. When children use short utterances, parents react with shorter utterances. The difference between the two groups is that parents of CI children use shorter utterances than parents of NH children, as was also seen with coarse tuning. This suggests that parents know unconsciously that it is important to adapt to the special needs of their hearing-impaired children by speaking with shorter utterances. It does not seem likely that parents consciously keep up with the words that their children know, but instead use implicit statistics to estimate the vocabulary size. Adults as well as children appear to have the ability to estimate the statistical regularities in their environment (Saffran et al., 1996, 1997). This could play a role in the parents' assessment of the level of development of their children. The finding that parents adapt their speech to the characteristics of their child, is in accordance with the findings of Kondaurova et al. (2013), who found that mothers are sensitive to the hearing status of their infants with CIs.

### Words in Isolation

An expected result of fine-tuning is that the frequency of single-word utterances with a particular word increases as the moment of the child's first production of that word approaches. Because words spoken in isolation are beneficial for word learning (Brent and Siskind, 2001), the frequency of words in isolation during the months before and after word birth was analyzed. The results showed that the incidence of words spoken in isolation are proportionate to the months from word birth. The closer to word birth, the more frequently that word occurs in a single-word utterance. Afterwards, words are embedded in more complex, longer utterances. Thus, children heard more isolated words when they were close to the first production of that word. A previous study by Brent and Siskind (2001) found that children learned words better in isolation. The frequency of hearing a word in isolation was a better predictor of word learning than the total frequency of that word's exposure. Other studies have also found a positive learning effect of words in isolation (Ninio, 2016; Swingley and Humphrey, 2018; Keren-Portnoy et al., 2019). In addition to words in isolation, shorter utterances are also helpful for learning words according to previous research (Swingley and Humphrey, 2018; Grimm et al., 2019). Single-word utterances may even be more beneficial to CI children, because the task of isolating a word in an utterance is easier when the word occurs in isolation as opposed to surrounded by other words. The current study showed that CI children heard even more isolated words than NH children. This suggests that Dutch-speaking parents take the specific characteristics of the child into account and consequently adapt their speech. These results add to the findings of Brent and Siskind (2001) and Swingley and Humphrey (2018). In the present study not only were the raw frequencies considered, but also the evolution of the incidence of isolated words. The present study shows that the occurrence of a word in parental single-word utterances relates to the actual acquisition (word birth) by the child.

In addition to the general longitudinal trend for words to occur more frequently in isolation as their first production by the child approached, differences between word classes were established in this respect. Nouns occurred much more frequently in isolation than verbs. Hence, these differences in frequency of word classes in a child's vocabulary may be partly explained by their differences in frequency as isolated words in parents' speech. Previous research showed that in specific languages, including Dutch, NH children and CI children exhibit a noun-bias (Gentner, 1982; Nott et al., 2009; Jung et al., 2020). This is also confirmed in the present study: of all the word births, 70% were nouns and 15% were verbs for the NH children, and this was 64 and 17%, respectively, for the CI children. Various explanations have been proposed for this phenomenon, such as the difference in frequency of nouns and verbs in IDS (Hart and Risley, 1995; Weizman and Snow, 2001). It has also been argued that nouns are conceptually simpler than verbs, thus easier to map to the world, which, again, makes them easier to learn. Furthermore, nouns are more frequent than verbs in short utterances and at the end of longer utterances (Goldfield, 1993). The present study found an additional phenomenon: nouns are much more frequent in single-word utterances. Thus, a characteristic of fine lexical tuning is that as word birth approaches, the utterance length decreases and the number of single-word utterances increases. Specifically for nouns, the number of single-word utterances is elevated, especially in comparison to other word classes. For verbs, the frequency is the lowest. Thus, if children benefit from single-word utterances in the early lexical stages, they can have much more advantage for nouns than for other word types. However, this is a language-specific feature, as a noun-bias is not found in all languages, so this could be different for other language groups.

Dutch-speaking parents' reducing their MLU around the time a word is first produced and the elevated frequency of isolated words, suggests that parents consider the child as an active interlocutor and take the specific characteristics of the child into account: word births are anticipated. This seems to hold a fortiori for a child with a hearing impairment: parents' MLU is lower and the number of single-word utterances is higher than in IDS addressed to NH children.

The findings reported in the current study require some additional qualifications. First of all, they are based on a corpus of Dutch-speaking parents and their children. This implies that the generalizability of the conclusions to parents and children speaking other language needs to be further investigated. It should be recalled in this respect that notwithstanding the fact that the noun bias was attested in various languages, the bias was not attested in Mandarin and Korean. Thus, the high frequency of nouns in single-word utterances may be restricted to a particular language group and, hence, not universally valid. Furthermore, the parents in the current study were from a mid-to-high SES background. May reports in the literature have shown social differences in language use of children as well as parents showing that differences in language may stem from social differences. Hence, the question remains if the results of the current study can be replicated in other SES environments.

## Conclusion

The current study demonstrated that Dutch-speaking parents of NH children and parents of CI children modify their utterances to the emergence of words in their children. Parents use both coarse tuning and fine lexical tuning. The MLU of parents of CI children is lower than that of parents of NH children, but both groups shorten their utterances as word birth approaches. Moreover, the significant increase of particular words used in isolation in IDS as words births are approaching, suggests that there is a specific role for single-word utterances in fine lexical tuning. Nouns specifically, are more frequently in isolation in IDS around the time of word birth and this may explain the finding that nouns are learned the earliest.

While both fine lexical tuning and the high occurrence of single-word utterances around word birth are seen in IDS to NH children and in IDS to CI children, some differences between the two groups were observed. This means that parents consider the child as interlocuter and take into account the specific characteristics of the child. This applies all the more for a child with hearing impairment, as the frequency of single-word utterances is higher and the MLU is lower, even when the children show a similar level of cumulative vocabulary.

## Data Availability Statement

The raw data supporting the conclusions of this article will be made available by the authors, without undue reservation.

## Ethics Statement

The studies involving human participants were reviewed and approved by Ethical Committee for the Social Sciences and Humanities of the University of Antwerp (EASHW_17_53). Written informed consent to participate in this study was provided by the participants' legal guardian/next of kin.

## Author Contributions

LO and SG contributed to conception, design of the study, and wrote sections of the manuscript. LO organized the database, performed the statistical analysis, and wrote the first draft of the manuscript. All authors contributed to manuscript revision, read, and approved the submitted version.

## Conflict of Interest

The authors declare that the research was conducted in the absence of any commercial or financial relationships that could be construed as a potential conflict of interest.
