# Supplementary material for: Tailoring the Input to Children's Needs: The Use of Fine Lexical Tuning in Speech Directed to Normally Hearing Children and Children With Cochlear Implants
Source: Front Psychol. 2021 Jun 17;12:676664. doi: 10.3389/fpsyg.2021.676664 (PMC8245684; doi:10.3389/fpsyg.2021.676664)
Supplement: Supplementary file 1 [file Table_1.docx]

**Appendix A. Overview of the cumulative vocabulary of children**

CI corpus

|  | **Months** | | | | | | | | | | | | | | | | | | |
| --- | --- | --- | --- | --- | --- | --- | --- | --- | --- | --- | --- | --- | --- | --- | --- | --- | --- | --- | --- |
| **Subject** | **6** | **7** | **8** | **9** | **10** | **11** | **12** | **13** | **14** | **15** | **16** | **17** | **18** | **19** | **20** | **21** | **22** | **23** | **24** |
| S1 |  |  |  |  |  |  |  |  | 0 | 0 | 0 | 0 | 0 | 0 | 0 | 9 | 9 | 16 | 18 |
| S2 | 0 | 0 | 0 | 0 | 0 | 0 | 0 | 0 | 0 | 0 | 1 | 1 | 4 | 5 | 7 | 10 | 23 | 32 | 48 |
| S3 |  |  |  |  |  | 0 | 0 | 0 | 0 | 0 | 0 | 0 | 0 | 0 | 3 | 7 | 14 | 21 | 34 |
| S4 |  |  |  |  |  |  |  |  |  |  |  |  |  | 1 | 3 | 4 | 4 | 4 | 5 |
| S5 |  |  |  |  |  |  |  |  | 0 | 0 | 0 | 0 | 0 | 0 | 0 |  | 0 | 4 | 13 |
| S6 |  |  |  |  |  |  |  |  |  |  |  |  |  | 0 | 3 | 9 | 21 | 55 |  |
| S7 |  |  |  |  |  |  |  |  | 0 | 0 | 1 | 3 | 13 | 23 |  | 39 | 60 | 71 | 89 |
| S8 |  | 0 | 0 | 0 | 0 | 0 | 0 | 0 | 0 | 2 | 6 | 8 | 12 | 19 | 33 | 46 | 61 | 83 | 104 |
| S9 |  |  |  |  |  |  |  |  |  |  |  |  |  |  |  | 1 | 1 | 1 | 6 |
| S10 |  |  |  |  |  | 0 | 0 | 0 | 1 | 8 | 9 | 18 | 47 | 69 | 116 | 181 | 237 |  | 327 |
|  | **25** | **26** | **27** | **28** | **29** | **30** | **31** | **32** | **33** | **34** | **35** | **36** | **37** | **38** | **39** | **40** | **41** | **42** | **43** |
| S1 | 32 | 47 | 47 | 70 | 91 | 131 | 139 | 162 | 183 | 223 | 242 | 277 | 334 |  |  |  |  |  |  |
| S2 | 86 | 106 | 113 | 157 | 192 | 240 | 264 | 322 | 351 |  |  |  |  |  |  |  |  |  |  |
| S3 | 46 | 80 | 103 | 127 | 177 | 222 | 267 | 318 |  |  |  |  |  |  |  |  |  |  |  |
| S4 | 5 | 5 | 5 | 6 |  |  |  |  |  | 37 | 82 | 99 | 129 | 144 | 168 | 168 | 183 | 219 | 253 |
| S5 | 19 |  |  |  |  |  |  |  |  |  |  |  |  |  |  |  |  |  |  |
| S6 | 101 | 111 | 151 | 180 | 215 | 253 | 287 | 325 |  |  |  |  |  |  |  |  |  |  |  |
| S7 | 102 | 129 | 164 | 184 | 231 | 266 | 309 | 346 |  |  |  |  |  |  |  |  |  |  |  |
| S8 | 124 | 149 |  | 193 | 238 | 278 | 311 |  |  |  |  |  |  |  |  |  |  |  |  |
| S9 | 8 | 24 | 45 | 53 | 73 | 89 | 107 | 135 | 158 | 210 | 246 | 260 | 288 | 318 |  |  |  |  |  |
| S10 |  |  |  |  |  |  |  |  |  |  |  |  |  |  |  |  |  |  |  |

NH corpus

| **Subject** | **6** | **7** | **8** | **9** | **10** | **11** | **12** | **13** | **14** | **15** | **16** | **17** | **18** | **19** | **20** | **21** | **22** | **23** | **24** |
| --- | --- | --- | --- | --- | --- | --- | --- | --- | --- | --- | --- | --- | --- | --- | --- | --- | --- | --- | --- |
| S11 | 0 | 0 | 0 | 0 | 0 | 0 | 0 | 0 | 1 | 12 | 14 | 26 | 48 | 58 | 78 | 115 | 149 | 191 | 235 |
| S12 | 0 | 0 | 0 | 0 | 0 | 0 | 1 | 6 | 11 | 25 | 41 | 52 | 63 | 72 | 85 | 102 | 144 | 178 | 233 |
| S13 | 0 | 0 | 0 | 0 | 0 | 0 | 0 | 0 | 0 | 4 | 9 | 20 | 41 | 61 | 105 | 145 | 178 | 241 | 284 |
| S14 | 0 | 0 | 0 | 0 | 0 | 0 | 0 | 3 | 10 | 25 | 49 | 62 | 92 | 122 | 147 | 183 | 247 | 309 |  |
| S15 | 0 | 0 | 0 | 0 | 0 | 1 | 2 | 3 | 4 | 6 | 6 | 9 | 23 | 28 | 41 | 75 | 103 | 136 | 178 |
| S16 | 0 | 0 | 0 | 0 | 0 | 0 | 0 | 0 | 0 | 1 | 3 | 6 | 12 | 19 | 25 | 32 | 49 | 61 | 65 |
| S17 | 0 | 0 | 0 | 0 | 0 | 0 | 0 | 0 | 0 | 0 | 1 | 5 | 17 | 24 | 37 | 63 | 103 | 139 | 195 |
| S18 | 0 | 0 | 0 | 0 | 0 | 0 | 1 | 2 | 2 | 2 | 5 | 8 | 18 | 30 | 39 | 56 | 81 | 136 | 178 |
| S19 | 0 | 0 | 0 | 0 | 0 | 0 | 0 | 0 | 0 | 7 | 21 | 48 | 66 | 104 | 145 | 195 | 262 | 311 |  |
| S20 | 0 | 0 | 0 | 0 | 0 | 0 | 0 | 0 | 0 | 2 | 4 | 6 | 11 | 27 | 40 | 62 | 100 | 140 | 208 |
| S21 | 0 | 0 | 0 | 0 | 0 | 0 | 0 | 0 | 0 | 0 | 1 | 3 | 5 | 13 | 23 | 33 | 48 | 70 | 108 |
| S22 | 0 | 0 | 0 | 0 | 0 | 0 | 0 | 0 | 0 | 0 | 1 | 7 | 14 | 21 | 32 | 43 | 54 | 65 | 77 |
| S23 | 0 | 0 | 0 | 0 | 0 | 0 | 0 | 0 | 5 | 14 | 18 | 30 | 45 | 76 | 95 | 199 | 259 | 308 | 346 |
| S24 | 0 | 0 | 0 | 0 | 0 | 0 | 3 | 9 | 10 | 10 | 16 | 29 | 41 | 73 | 93 | 124 | 153 | 210 | 284 |
| S25 | 0 | 0 | 0 | 0 | 0 | 0 | 0 | 0 | 0 | 0 | 8 | 8 | 8 | 13 | 16 | 29 | 57 | 93 | 133 |
| S26 | 0 | 0 | 0 | 0 | 0 | 1 | 3 | 8 | 8 | 13 | 15 | 18 | 19 | 21 | 23 | 27 | 31 | 54 | 89 |
| S27 | 0 | 0 | 0 | 0 | 0 | 0 | 4 | 5 | 12 | 18 | 23 | 27 | 41 | 57 | 80 | 100 | 148 | 184 | 227 |
| S28 | 0 | 0 | 0 | 0 | 0 | 0 | 1 | 4 | 11 | 19 | 27 | 35 | 50 | 69 | 94 | 133 | 185 | 243 | 303 |
| S29 | 0 | 0 | 0 | 0 | 0 | 0 | 0 | 2 | 5 | 6 | 15 | 20 | 27 | 34 | 38 | 95 | 133 | 158 | 192 |
| S30 | 0 | 0 | 0 | 0 | 0 | 0 | 2 | 5 | 8 | 14 | 18 | 31 | 47 | 66 | 86 | 106 | 131 | 161 | 199 |
| S31 | 0 | 0 | 0 | 0 | 0 | 0 | 0 | 0 | 2 | 3 | 7 | 16 | 24 | 40 | 55 | 93 | 143 | 209 | 278 |
| S32 | 0 | 0 | 0 | 0 | 0 | 0 | 4 | 4 | 7 | 12 | 20 | 31 | 40 | 44 | 66 | 82 | 110 | 172 | 215 |
| S33 | 0 | 0 | 0 | 0 | 0 | 0 | 2 | 8 | 14 | 18 | 32 | 46 | 64 | 85 | 110 | 148 | 174 | 212 | 259 |
| S34 | 0 | 0 | 0 | 0 | 1 | 1 | 1 | 14 | 19 | 26 | 39 | 53 | 98 | 116 | 148 | 194 | 214 | 227 | 323 |
| S35 | 0 | 0 | 0 | 0 | 0 | 0 | 1 | 4 | 5 | 8 | 12 | 20 | 32 | 48 | 64 | 74 | 99 | 112 | 137 |
| S36 | 0 | 0 | 0 | 0 | 0 | 0 | 0 | 0 | 2 | 4 | 4 | 5 | 10 | 14 | 16 | 19 | 35 | 45 | 56 |
| S37 | 0 | 0 | 0 | 0 | 0 | 0 | 0 | 1 | 2 | 9 | 15 | 24 | 39 | 77 | 118 | 193 | 236 | 279 | 319 |
| S38 | 0 | 0 | 0 | 0 | 0 | 1 | 3 | 6 | 7 | 11 | 25 | 32 | 37 | 47 | 56 | 66 | 83 | 119 | 156 |
| S39 | 0 | 0 | 0 | 0 | 0 | 0 | 0 | 0 | 1 | 6 | 7 | 8 | 13 | 21 | 42 | 52 | 75 | 107 | 144 |
| S40 | 0 | 0 | 0 | 0 | 0 | 0 | 0 | 0 | 2 | 2 | 8 | 14 | 27 | 40 | 51 | 65 | 83 | 100 | 131 |
