# Supplementary material for: Tailoring the Input to Children's Needs: The Use of Fine Lexical Tuning in Speech Directed to Normally Hearing Children and Children With Cochlear Implants
Source: Front Psychol. 2021 Jun 17;12:676664. doi: 10.3389/fpsyg.2021.676664 (PMC8245684; doi:10.3389/fpsyg.2021.676664)
Supplement: Supplementary file 2 [file Table_2.docx]

**Appendix B. Statistical Models**

**Table 1 Parameters estimates of MLU of utterances with target words**

| Effect | Estimate | SE | t value | *p* |
| --- | --- | --- | --- | --- |
| *Fixed Parameters* |  |  |  |  |
| Intercept | 4.36 | 0.28 | 15.48 | <0.0001*** |
| Months from word birth | 0.14 | 0.028 | 4.99 | <0.0001*** |
| Quadratic months from word birth | -0.02 | 0.002 | -8.21 | <0.0001*** |
| Cubic months from word birth | 0.0005 | 0.00005 | 9.21 | <0.0001*** |
| Cumulative vocabulary | 0.001 | 0.0003 | 3.30 | 0.001** |
| Hearing status [NH] | 0.64 | 0.23 | 2.83 | 0.005** |
| Word class [Adverb] | 0.47 | 0.20 | 2.28 | 0.02* |
| Word class [Noun] | -0.39 | 0.15 | -2.63 | 0.008** |
| Word class [Verb] | 0.14 | 0.16 | 0.83 | 0.40 |
|  |  |  |  |  |
| *Random Parameters* |  |  |  |  |
| s^2^ Word (intercept) | 0.74 | 0.07 |  |  |
| s^2^ Word * months from word birth | 0.002 | 0.0007 |  |  |
| s^2^ Child (intercept) | 0.56 | 0.19 |  |  |
| s^2^ Child * months from word birth | 0.002 | 0.0007 |  |  |
| s^2^ Residual | 3.67 | 0.05 |  |  |

p<0.05*

p<0.01**

p<0.001***

**Table 2 Parameters estimates of frequencies of isolated words**

| Effect | Estimate | SE | t value | *p* |
| --- | --- | --- | --- | --- |
| *Fixed Parameters* |  |  |  |  |
| Intercept | 0.79 | 0.12 | 6.53 | <0.0001*** |
| Months from word birth | -0.14 | 0.03 | -4.73 | <0.0001*** |
| Quadratic months from word birth | 0.02 | 0.002 | 8.01 | <0.0001*** |
| Cubic months from word birth | -0.0005 | 0.00005 | -10.08 | <0.0001*** |
| Hearing status [NH] | -0.15 | 0.04 | -3.63 | 0.0003** |
| Word class [Verb] | -0.41 | 0.18 | -2.29 | 0.02* |
| Word class [Adjective] | -0.04 | 0.28 | -0.15 | 0.88 |
| Word class [Adverb] | -0.24 | 0.15 | -1.60 | 0.11 |
| Months from word birth * Word class [Verb] | 0.09 | 0.05 | 1.96 | 0.05 |
| Months from word birth * Word class [Adjective] | -0.07 | 0.07 | -0.98 | 0.33 |
| Months from word birth * Word class [Adverb] | 0.07 | 0.04 | 1.87 | 0.06 |
| Quadratic months from word birth * Word class [Verb] | -0.01 | 0.003 | -3.12 | 0.002** |
| Quadratic months from word birth * Word class [Adjective] | 0.002 | 0.005 | -0.40 | 0.69 |
| Quadratic months from word birth * Word class [Adverb] | -0.009 | 0.003 | -3.14 | 0.002** |
| Cubic months from word birth * Word class [Verb] | 0.0003 | 0.00008 | 3.84 | 0.001** |
| Cubic months from word birth * Word class [Adjective] | -0.000003 | 0.0001 | 0.02 | 0.98 |
| Cubic months from word birth * Word class [Adverb] | 0.0003 | 0.0001 | 3.87 | 0.001** |
|  |  |  |  |  |
| *Random Parameters* |  |  |  |  |
| s^2^ Child (intercept) | 0.017 | 0.005 |  |  |
| s^2^ Child * months from word birth | 0.00001 | 0.00004 |  |  |
| s^2^ Residual | 0.12 | 0.004 |  |  |

p<0.05*

p<0.01**

p<0.001***
